# Diverging Paths: Longitudinal and Reciprocal Associations Between Two Fundamental Motor Skill Assessments in Preschoolers

**DOI:** 10.1111/cch.70295

**Published:** 2026-05-18

**Authors:** E. Kipling Webster, Katherine E. Spring, Justin X. Moore, Dimetrius Brandon, Amanda E. Staiano

**Affiliations:** ^1^ Department of Kinesiology, Recreation, and Sport Studies University of Tennessee Knoxville Tennessee USA; ^2^ Pennington Biomedical Research Center Baton Rouge Louisiana USA; ^3^ Center for Health Engagement, and Transformation, Department of Behavioral Science, College of Medicine University of Kentucky Lexington Kentucky USA

**Keywords:** MABC‐2, motor development, preschool, TGMD‐3

## Abstract

**Background:**

Fundamental motor skills (FMS) are critical for child health and development and are commonly assessed using either process‐oriented assessment or product‐oriented assessments. Although previous studies have examined cross‐sectional relationships between these assessments, little is known about their longitudinal and reciprocal associations. Therefore, the purpose of this study was to examine the longitudinal and reciprocal relationships of two FMS assessments (Test of Gross Motor Development–3rd edition [TGMD‐3] and Movement Assessment Battery for Children–2nd edition [MABC‐2]) in preschool‐age children.

**Methods:**

A diverse sample of 117 children (*Mage* = 3.4 years) completed both assessments at baseline, with 72 completing follow‐up. Spearman correlations were conducted to assess cross‐sectional relationships, and linear mixed‐effects models (LMMs) were used to model longitudinal associations, adjusting for race, BMI, SES, screen time, moderate‐to‐vigorous physical activity (MVPA) and childcare centre.

**Results:**

At baseline, significant positive associations were found between TGMD‐3 and MABC‐2 total and subscale percentile scores (*r*_s_ = 0.20–0.41, all *p* < 0.05), with the strongest correlations observed between TGMD‐3 total and MABC‐2 total scores (*r*_s_ = 0.41). However, no significant correlations were found at the Year 2 follow‐up nor cross‐predictive longitudinal associations (all *p* > 0.05). LMMs revealed that MABC‐2 scores at baseline significantly predicted TGMD‐3 scores at baseline (*β* = 0.57), explaining 14.2% of the variance, but not at follow‐up (*β* = −0.02). Each assessment significantly predicted its own follow‐up performance: TGMD‐3 (*β* = 0.42; 27% variance explained) and MABC‐2 (*β* = 0.52; 20.3% variance explained).

**Conclusions:**

These findings suggest that TGMD‐3 and MABC‐2 measure distinct constructs of FMS performance that may diverge as children develop. The study highlights the importance of selecting FMS assessments based on the developmental stage, purpose and context and emphasizes the need for longitudinal research to better understand how early FMS influence long‐term outcomes.

**Trial Registration:**

ClinicalTrials.gov ID: NCT02751788

## Summary

- TGMD‐3 and MABC‐2 assess different dimensions of fundamental motor skills, and their divergence over time suggests that they should not be used interchangeably in longitudinal research or practice.
- The lack of cross‐predictive validity between assessments over time underscores the importance of choosing FMS tools that align with a child's development stage and the specific goals of assessment (e.g., screening vs. intervention planning).
- The study highlights a gap in understanding how different motor skill domains evolve and interact over time; more longitudinal studies are needed to inform developmental trajectories and intervention timing.
- Researchers and policymakers should consider supporting the use of multiple, complementary FMS assessments in early childhood settings to ensure a holistic understanding of motor competence and to guide targeted interventions.

Fundamental motor skills (FMS), which consist of gross motor skills, fine motor skills and stability skills that develop in early childhood, are an essential component of healthy development and are considered the building blocks for more advanced movements (Clark and Metcalfe 2002; Logan et al. 2018). Gross motor skills are comprised of both locomotor skills and object control skills, which include both object manipulation and object projection tasks (Clark and Metcalfe 2002). Competency in FMS allows children to independently navigate and interact with their environment. Higher competence in FMS is associated with a plethora of benefits for lifelong health including higher levels of physical activity (Logan et al. 2015; Robinson et al. 2015), health‐related physical fitness (Cattuzzo et al. 2016) and decreased overweight/obesity (Barnett et al. 2022). Therefore, accurate assessment of these skills is a critical piece in understanding their influence on other health behaviours and how FMS develop in early childhood (Tamplain et al. 2020).

An objective way to measure FMS, which is deemed both accurate and a direct estimate of motor competence, is through evaluation by either process‐ or product‐oriented assessments (Bardid et al. 2019). Process‐oriented assessments measure performance elements such as how the arms or legs are moving during a specific movement pattern and whether these are appropriate for skill execution. A frequently used process‐oriented FMS assessment is the Test of Gross Motor Development (TGMD; Tamplain et al. 2020; Ulrich 1985, 2000; Ulrich 2019). The TGMD‐3rd edition (TGMD‐3) examines 13 FMS, either locomotor or object control (i.e., ball) skills, in children 3–11 years of age. By contrast, product‐oriented assessments measure the outcome of a movement pattern, so rather than focusing on *how* a ball is thrown, these assessments consider throwing speed or accuracy instead. A frequently used product‐oriented assessment is the Movement Assessment Battery for Children (MABC; Henderson and Sugden 1992; Henderson et al. 2007; Tamplain et al. 2020), which measures manual dexterity, ball skills and balance in children 3–16 years of age. Generally, assessments are selected based on the purpose, test population, testing environment and background of those conducting the tests (Bardid et al. 2019; Cools et al. 2009).

Previous research has examined the relationship between process‐ and product‐oriented assessments in children and found varying associations based on the specific assessments used (e.g., TGMD‐2 and MABC‐2nd edition [MABC‐2]) and what age groups were examined. There are several studies that have examined the cross‐sectional associations between the TGMD‐2 and MABC‐2. In preschool‐age children, low‐to‐moderate correlations were found between the subscales of the TGMD‐2 and MABC‐2; total performance was not significantly associated (Logan et al. 2011). Also, Valentini et al. (2015) found low associations between the two assessments in children 4–11 years of age. By contrast, in another study comparing four different types of assessments, three process‐oriented assessments (TGMD‐2; Get Skilled, Get Active; and Development Sequences) and one product‐oriented measure (throwing speed, hop and jump distance), children aged 4–11 years had moderate‐to‐strong correlations between assessments (Logan et al. 2017). Since most studies and interventions rely only on one FMS assessment, understanding how different assessments relate or differentiate to one another may help explain associations between FMS and other health outcomes. To better interpret both significant and null findings related to health behaviours (e.g., physical activity) or intervention effectiveness, it is also important to consider naturalistic findings over time. For example, Palmer et al. (2021) examined intervention effectiveness using the TGMD‐3 and six individual product scores to evaluate changes in FMS over an intervention. Results showed improvements on the skills evaluated from a process‐oriented assessment (i.e., TGMD‐3), but not on the aggregate product score measure, highlighting that these different evaluations of FMS did not equally reflect intervention efficacy for this particular programme. Although studies have examined cross‐sectional relationships, few studies have examined longitudinal along with reciprocal associations.

Considering the importance FMS plays in early childhood health, understanding FMS by examining both process‐ and product‐oriented aspects of movement patterns can provide a more holistic way of viewing FMS (Robinson et al. 2015). Similarly, measuring FMS across multiple time points can illuminate how these FMS change over time and how early patterns may influence or predict later performance (Tamplain et al. 2020). Limited information is available on how these assessments relate to each other and over time as previous literature has been primarily cross‐sectional in nature. Therefore, the purpose of this study was to examine the longitudinal and reciprocating associations of two FMS assessments (TGMD‐3 and MABC‐2) in preschool‐age children.

## 1 Methods

*Pause & Play* was a prospective observational cohort study that included a natural experiment that examined the implementation and effectiveness of new state childcare centre policies regarding physical activity and electronic device use on children's physical activity and related health behaviours over 1 year (Kracht et al. 2020; Staiano, Allen, et al. 2018; Staiano, Webster, et al. 2018; Webster et al. 2019). The present analysis includes both baseline and 1‐year follow‐up data from this larger study. The study protocol was approved by Pennington Biomedical Research Center's Institutional Review Board. Parent/legal guardian provided written consent, and verbal child assent was obtained prior to data collection.

### 1.1 Procedures

Full study details may be found elsewhere (Kracht et al. 2020; Webster et al. 2019). In short, preschools were stratified by government childcare assistance from one geographical region in the south‐eastern portion of the United States and randomly selected to participate. Ten childcare centres agreed to participate at the study onset, and nine continued participation 1 year later at follow‐up. Upon receiving written consent from the director, parents of children attending the participating schools were invited to participate, and parents/legal guardians provided written consent for their child to participate. All measures were explained in child‐friendly terms, and children were allowed to refuse participation or stop at any time. All children aged 3–4.9 years were invited to participate if they were enrolled full‐day at a participating childcare centre and had intentions to stay 1 year later for follow‐up.

Children were assessed at each childcare centre in small groups. Participants completed the two FMS assessments in a counterbalanced order. The assessments included the TGMD‐3 and the MABC‐2. Children completed each assessment in small groups of three to four children. The TGMD‐3 was conducted as a group, whereas children completed the MABC‐2 individually but maintained the small group size and rotated through stations completing each task. FMS testing for each assessment took place on separate days. Centres were contacted approximately 1 year later, after the original data collection took place, to conduct the same assessments using the same protocol.

### 1.2 TGMD–3rd Edition

Participants completed the TGMD‐3 (Ulrich 2019), a reliable and valid, process‐oriented assessment of FMS in children 3 years up to 11 years of age (Webster and Ulrich 2017). The assessment consists of 13 different FMS, all gross motor skills, that are categorized into two subscales, locomotor skills and ball skills (i.e., object control skills). There are a total of six locomotor control skills (run, gallop, hop, skip, horizontal jump and slide) and seven ball skills (dribble, two hand strike with bat, one hand strike with paddle, underhand throw, overhand throw, catch and kick). Each participant watched a trained administrator demonstrate the skill and then was allowed three attempts on their own. The first attempt was a practice trial, whereas the second and third attempt were scored for evaluation. Each skill is evaluated by three to five performance criteria, for example, ‘pushes the ball with fingertips’ for dribbling. These performance criteria are evaluated by a trained administrator for their presence or absence in each formal trial. For instance, if a participant correctly pushed the ball with their fingertips when performing the stationary dribble, they would receive a score of 1 for that criterion. If this was done incorrectly, they would receive a score of 0. The TGMD‐3 raw scores range from 0 to 54 for ball skills and from 0 to 46 for locomotor control skills with a total raw score possible being 100 (Ulrich 2019). A higher score represents a better performance on the assessment. In addition, raw scores were transformed into age equivalents, standard scores or percentiles for a description of their performance compared to peers of the same age and sex using the TGMD‐3 normative data (Ulrich 2019). TGMD‐3 performance was filmed and later analysed by two trained researchers according to manual guidelines who established 95% reliability with an expert coder prior to coding and had interrater reliability of 98% for the total TGMD‐3 scores.

### 1.3 MABC–2nd Edition

Participants completed the MABC‐2 (Henderson et al. 2007), a reliable and valid, product‐oriented assessment of FMS, both fine and gross motor skills, in children aged 3–16 years. This assessment consists of eight FMS that are categorized into three subscales: manual dexterity, aiming and catching and balance. Based on the product‐oriented nature of this assessment, there are different age bands that assess the same skills in incrementally more challenging, age‐appropriate ways. The present study used Age Band 1 for 3‐ to 6‐year‐old children. The manual dexterity subscale includes three tasks: posting coins, threading beads and a drawing trail task. The aiming and catching subscale involves catching a beanbag and throwing a beanbag toward a target. The balance subscale includes one‐leg balance, walking on a line with the heels raised and jumping. Each FMS activity is scored using product‐oriented measures, such as the time it takes to successfully thread all beads onto a string (threading beads) or the number of consecutive jumps completed across a set of mats (jumping). These scores are then transformed into standard scores and percentiles based on the child's age and sex. Higher scores represent a better performance on the assessment. Each performance was scored live by trained research staff according to manual guidelines.

### 1.4 Covariates

Height and weight were measured by trained staff to calculate body mass index (BMI) percentile (Kuczmarski et al. 2000). Physical activity was measured using an accelerometer (Actigraph G3TX+) on the child's right hip for 7 days, 24 h/day; accelerometry data collection did not overlap with the FMS assessment days. ActiLife Software version 5.6 (ActiGraph) was used to process accelerometer data to calculate wear time and duration of activity. Data were processed using 15‐s epochs, and valid wear time was considered to be at least 10 h of wear time for at least 3 days (Kracht et al. 2020; Webster et al. 2019). Nonwear time was established by 30 min of continuous 0 count per min (cpm), except during sleep time. Age‐appropriate cutpoints (Pate et al. 2006) were applied to determine sedentary behaviour and physical activity intensities. Parents completed a questionnaire, which included questions on demographics, family income that was used as a proxy for socioeconomic status (SES), parental education and their child's screen time habits (i.e., average minutes per day of by screen device; whether TV is located in child's bedroom).

## 2 Statistical Analyses

For descriptive analysis, the number of participants with relative frequencies/proportions or mean and standard errors (SE) for categorical and continuous variables were calculated, respectively. Spearman's rho correlations were conducted to examine the concurrent validity between TGMD‐3 and MABC‐2 total and subscale percentile scores across Year 1 and Year 2 not controlling for any confounding variables. Then, we estimated the mean MABC‐2 and TGMD‐3 percentile scores stratified for several child characteristics including sex, race, ethnicity, annual household income, quartiles of moderate‐to‐vigorous physical activity (MVPA) (based on accelerometry) and presence of TV in the child's bedroom (based on parent report).

Linear mixed‐effects models (LMMs) were performed to examine the relationships between multiple factors and two primary follow‐up outcomes: (1) TGMD‐3 and (2) MABC‐2. LMMs extend traditional general linear models by incorporating both fixed effects, which estimate population‐average relationships, and random effects, which account for within‐subject or cluster‐level correlation arising from repeated or nested observations (Laird and Ware 1982). This framework allows for valid estimation in the presence of correlated data and unbalanced designs, while using all available observations under maximum likelihood assumptions. Compared with ordinary least squares (OLS) regression, LMMs provide more appropriate standard errors and inference when observations are not independent and are especially useful for longitudinal or clustered data structures (Laird and Ware 1982). Tables 2, 3, 4 examined longitudinal and concurrent associations between percentile scores on the MABC‐2 and TGMD‐3. In Table 2, we assessed cross‐sectional associations between MABC‐2 and TGMD‐3 measured at baseline and follow‐up, hypothesizing that higher MABC‐2 scores would be positively associated with higher TGMD‐3 scores at each corresponding time point. In Table 3, we evaluated whether baseline TGMD‐3, baseline MABC‐2 and change in MABC‐2 from baseline to follow‐up were associated with TGMD‐3 at follow‐up; we hypothesized that higher baseline scores and greater improvement in MABC‐2 would predict higher follow‐up TGMD‐3 scores. In Table 4, we examined whether baseline MABC‐2, baseline TGMD‐3 and change in TGMD‐3 were associated with MABC‐2 at follow‐up, hypothesizing that higher baseline motor scores and greater improvement in TGMD‐3 would be associated with higher follow‐up MABC‐2 scores.

Analysis of correlated data using OLS methods may result in artificially low variance and low *p*‐values (Hanley et al. 2003; Zeger and Liang 1986). We accounted for correlated data from participants clustered within childcare centres and repeated outcomes (at baseline and follow‐up). Univariable models examining the independent association between factors with measures of TGMD‐3 and MABC‐2 at follow‐up. Multivariable modelling accounted for potential confounding of race/ethnicity, BMI, SES, childcare centre, screen time and MVPA. Additionally, independently fit generalized linear models were used to estimate coefficients of determination, that is, *R* ^2^ values, for each variable in relation to primary outcomes. SAS software version 9.4 (Cary, NC, USA) was used for all analyses.

## 3 Results

For this analysis, 117 children had complete data at baseline, and 72 participants had complete data 1 year later at follow‐up. Dropouts were primarily related to changing childcare centre or incomplete measures at follow‐up. Descriptive characteristics can be found in Table 1. On average, participant follow‐up was almost 1 year later (*M* = 363.3 days; SE = 2.5), and children were on average 3.4 years of age (SE = 0.1) at baseline measurements. SES was diverse in this sample and most children were female (53%) and almost half the children were Black/African American (48.7%). Nineteen percent of the sample were overweight and 10.3% of children had obesity.

**TABLE 1:** Characteristics of 117 child participants of the Pause & Play study.

Characteristic	*N* (%) or mean (SE^a^)	Mean MABC^b^ (SE) at baseline	Mean TGMD‐3^c^ (SE) at baseline
Mean follow‐up (SE), in days	363.3 (2.5)		
Overall			
Mean age (SE), in years	3.36 (0.05)		
Gender			
Female	62 (53.0)	7.5 (0.3)	18.8 (0.4)
Male	55 (47.0)	7.8 (0.4)	19.6 (0.6)
Race			
Asian	10 (8.6)	9.0 (1.2)	16.8 (1.3)
Black	57 (48.7)	7.5 (0.3)	20.0 (0.5)
Other	4 (3.4)	6.8 (0.8)	20.3 (1.4)
White	46 (39.3)	7.7 (0.4)	18.6 (0.5)
Ethnicity			
Hispanic or Latinx	4 (3.4)	8.3 (0.3)	20.3 (1.1)
Non‐Hispanic/Latinx	113 (96.6)	7.6 (0.2)	19.2 (0.3)
Mean BMI percentile (SE)	65.51 (2.63)		
Normal weight	82 (70.7)		
Overweight	22 (19.0)		
Obese	12 (10.3)		
Annual household income			
< $30 000	41 (35.0)	7.5 (0.3)	19.8 (0.6)
$30 000–69 999	9 (7.7)	6.7 (0.9)	19.8 (1.3)
$70 000–109 999	16 (13.7)	7.7 (0.8)	18.1 (0.7)
$110 000+	34 (29.1)	7.7 (0.4)	18.3 (0.7)
Not reported	17 (14.5)	8.5 (0.7)	20.2 (0.8)
Mean MVPA (SE), in minutes	105.7 (3.9)		
Quartiles of MVPA			
1st quartile (36.50–81.25)	20 (17.1)	7.0 (0.6)	18.3 (0.8)
2nd quartile (81.25–100.13)	22 (18.8)	8.0 (0.7)	18.9 (0.6)
3rd quartile (100.13–125.38)	21 (18.0)	8.2 (0.6)	18.3 (0.7)
4th quartile (125.38–226.00)	20 (17.1)	7.6 (0.5)	19.1 (0.9)
Missing	34 (29.1)	7.5 (0.4)	20.5 (0.6)
TV in bedroom			
No	62 (53.0)	7.8 (0.3)	18.7 (0.5)
Yes	55 (47.0)	7.5 (0.4)	19.7 (0.5)
Mean screen time per day (SE), in minutes	334.1 (24.8)		
Mean television time per day (SE), in minutes	119.5 (7.1)		
Mean computer time per day (SE), in minutes	83.4 (9.1)		
Mean video games time per day (SE), in minutes	70.7 (8.6)		
Mean smartphone time per day (SE), in minutes	75.7 (7.5)		
Mean tablet time per day (SE), in minutes	93.0 (8.3)		

^a^ SE = standard error.

^b^ MABC‐2 standard score, Movement Assessment for Battery for Children–2nd edition.

^c^ TGMD‐3 standard score, Test of Gross Motor Development–3rd edition.

**FIGURE 1:**
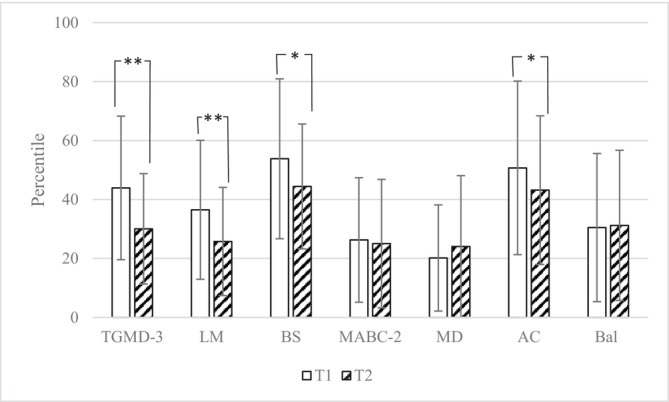
TGMD‐3 and MABC‐2 total and subscale percentiles for baseline and follow‐up, among 72 participants. *Note:* **p < 0.001, *p < 0.05; AC = aiming and catching subscale; Bal = balance subscale; BS = ball skill subscale; LM = locomotor subscale; MD = manual dexterity subscale.

Means and standard deviations for TGMD‐3, MABC‐2, and subscale percentiles across both time points can be found in Figure 1. There were significant differences from baseline to follow‐up for the TGMD‐3 total percentile score, as well as the locomotor and ball skills subscales in this study. Baseline percentile scores for both the TGMD‐3 and MABC‐2 decreased from baseline to follow‐up 1 year later. For the MABC‐2, the total and subscale scores decreased over 1 year, but not significantly, except for the manual dexterity subscale that slightly increased but was not a statistically significant change.

At baseline (Year 1), Spearman's correlations were conducted to examine associations between TGMD‐3 subscales (ball skills and locomotor) and total percentile scores and MABC‐2 subscales (manual dexterity, aiming and catching and balance) and total percentile scores. TGMD‐3 ball skills had significant positive correlations with MABC‐2 aiming and catching (*r*_s_ = 0.32, *p* < 0.001) and total percentile scores (*r*_s_ = 0.26, *p* = 0.004). No significant associations were found with balance or manual dexterity (all *p* > 0.05). TGMD‐3 locomotor skills had significant positive correlations with MABC‐2 balance (*r*_s_ = 0.35, *p* < 0.001), aiming and catching (*r*_s_ = 0.20, *p* = 0.035) and total percentile scores (*r*_s_ = 0.36, *p* < 0.001). No significant correlation was found between TGMD‐3 locomotor scores and manual dexterity (*p* > 0.05). The TGMD‐3 total scores were significantly correlated with MABC‐2 balance (*r*_s_ = 0.34, *p* < 0.001), aiming and catching (*r*_s_ = 0.34, *p* < 0.001) and total percentile scores (*r*_s_ = 0.41, *p* < 0.001), whereas no significant association was found with manual dexterity (*p* > 0.05). Looking at the longitudinal associations, no statistically significant associations were found in Year 2 between TGMD‐3 and MABC‐2 total scores or subscales or total percentile scores (all *p* > 0.05).

### 3.1 Linear Mixed Models

MABC‐2 scores at baseline were significantly associated with TGMD‐3 scores at baseline (Model 1: *b* = 0.57, 95% CI = 0.30, 0.85) accounting for race, ethnicity, BMI, SES, childcare centre, screen time and MVPA (Table 2) and explained 14.2% of the variation. There was no significant association between MABC‐2 scores and TGMD‐3 scores at follow‐up (Model 1: *b* = −0.02, 95% CI = −0.42, 0.37) when accounting for race, ethnicity, BMI, SES, childcare centre, screen time and MVPA.

**TABLE 2:** Concurrent associations between TGMD‐3 and MABC‐2 using linear mixed models.

	No. of participants	*β* (95% CI)	*β* ^a^ (95% CI)	*R* ^2^ (% variance explained^b^)
		Association with TGMD‐3 at baseline		
Variable				
MABC‐2 at baseline	117	0.52 (0.28, 0.76)	0.57 (0.30, 0.85)	14.2%
		Association with TGMD‐3 at follow‐up		
Variable				
MABC‐2 at time 2	72	0.08 (−0.21, 0.37)	−0.02 (−0.42, 0.37)	0.4%

^a^ Model 1: Adjusted for race/ethnicity, BMI, SES, childcare centre, screen time and MVPA.

^b^ Variance explained by independent variable alone using linear regression model for estimation.

TGMD‐3 scores at baseline had a positive association with TGMD‐3 scores at follow‐up (Model 1: *b* = 0.42, 95% CI = 0.24, 0.61) after controlling for potential confounders. TGMD‐3 baseline performance accounted for about 27% of the variation in TGMD‐3 at follow‐up (Table 3). MABC‐2 percentile scores at baseline and change in MABC‐2 scores across the two time points were not significantly associated with TGMD‐3 scores at follow‐up after controlling for potential confounders and only explained 4.4% and 4.7% of the variance, respectively.

**TABLE 3:** Relationship between each factor (TGMD‐3, MABC‐2 and ΔMABC‐2) on TGMD‐3 at follow‐up, among 72 child participants using linear mixed models.

	*β* (95% CI)	*β* ^a^ (95% CI)	*R* ^2^ (% variance explained^b^)
Variable			
	TGMD‐3 percentiles (at follow‐up)		
TGMD‐3 Per at baseline	0.40 (0.25, 0.56)	0.42 (0.24, 0.61)	27.3%
MABC‐2 Per at baseline	0.19 (−0.02, 0.39)	0.04 (−0.23, 0.31)	4.4%
Δ MABC‐2 Per^c^	−0.18 (−0.37, 0.01)	−0.13 (−0.40, 0.13)	4.7%

Abbreviation: Per = percentile scores.

^a^ Model 1: Adjusted for race/ethnicity, BMI, SES, childcare centre, screen time and MVPA.

^b^ Variance explained by independent variable alone.

^c^ Δ represents the change from Time 1 (baseline) and follow‐up.

MABC‐2 scores at baseline were positively associated with MABC‐2 scores at follow‐up (Model 1: *b* = 0.52, 95% CI = 0.21, 0.83) and accounted for 20.3% of the variation in MABC‐2 percentiles during follow‐up visit (Table 3). TGMD‐3 percentile scores at baseline and change in TGMD‐3 scores across the two time points were not significantly associated with MABC‐2 scores at follow‐up after controlling for potential confounders, and both only explained 0.01% of the variance (Table 4).

**TABLE 4:** Relationship between each factor (TGMD‐3, MABC‐2 and ΔTGMD‐3) and MABC‐2 at follow‐up, among 72 child participants using linear mixed models.

	*β* (95% CI)	*β* ^a^ (95% CI)	*R* ^2^ (% variance explained^b^)
Variable			
	MABC‐2 percentiles (follow‐up)		
MABC‐2 Per at baseline	0.46 (0.24, 0.68)	0.52 (0.21, 0.83)	20.3%
TGMD‐3 Per at baseline	0.02 (−0.19, 0.23)	0.09 (−0.22, 0.41)	0.01%
Change in TGMD‐3 Per	−0.04 (−0.28, 0.20)	−0.28 (−0.67, 0.12)	0.01%

^a^ Model 1: Adjusted for race/ethnicity, BMI, SES, childcare centre, screen time and MVPA.

^b^ Variance explained by independent variable alone.

### 3.2 Sensitivity Analysis

We conducted a comparison of baseline characteristics between participants who completed follow‐up (*n* = 72) and those lost to follow‐up (*n* = 45). Children who did not complete follow‐up were more likely to be older at baseline (mean age: 3.53 vs. 3.25 years), identify as Black race (64.4% vs. 38.9%), have higher baseline BMI percentile (67.5 vs. 57.7) and come from households with annual income < $30 000 (51.1% vs. 25.0%), compared with children who completed follow‐up. Those lost to follow‐up also had higher baseline MVPA (116.5 vs. 99.5 min/day), greater total screen time (415.3 vs. 283.3 min/day), higher television viewing time (137.3 vs. 108.2 min/day) and higher computer use (105.0 vs. 64.6 min/day); see Table S1 for full details.

## 4 Discussion

The aim of the current study was to examine the concurrent and longitudinal associations of two FMS assessments in preschool‐aged children. The findings of the current study support the body of literature (Logan et al. 2017; Palmer et al. 2021; Re et al. 2018) comparing process‐ and product‐oriented FMS assessments cross sectionally. To date, the general consensus is that process‐ and product‐oriented are not interchangeable regarding performance measurement in preschool‐aged children. The present study expands these findings, particularly from a longitudinal perspective, where the results from this investigation provide new insight into how these relationships seemingly weaken over time, which suggest caution should be taken when using assessments interchangeably or ensuring you are aligning the appropriate assessment for your research question. When children were 3–4 years old we found a significant association, albeit small amount of overall variance explained, between the MABC‐2 and TGMD‐3 (14.2% of variance explained), but this dissipated 1 year later by ages 4–5 years (0.4% of variance explained). This weakening association over time may reflect divergent developmental trajectories of the motor domains captured by each tool. Notably, although percentile scores generally declined across both assessments over the 1‐year period, manual dexterity was the only subscale that showed a slight increase. Because TGMD‐3 emphasizes gross motor activities (e.g., running, jumping and throwing) and MABC‐2 includes more fine motor skills examining manual dexterity, it is possible that as children age, their FMS development becomes more domain‐specific—resulting in less overlap between TGMD‐3 and MABC‐2. This asynchronous development may contribute to the lack of cross‐predictive validity observed and suggests that relying on a single tool may not capture the full picture of FMS performance during this stage of development.

Descriptively, the sample was diverse in both race and SES, and children were, on average, 3.4 years old at baseline with a follow‐up occurring approximately 1 year later. The percentile scores for TGMD‐3 significantly declined over the 1‐year period, in both the locomotor and ball skills subscales. In contrast, MABC‐2 scores remained relatively stable across most subscales, except for a significant decline in aiming and catching. It is important to note these results reflect percentile scores, not raw scores, which means they are using a normative sample to compare performance. Because the normative sample advances in performance over time, a child who remains the same begins to decrease in percentile score as they deviate from the performance of other children their age. Additionally, these findings provide further evidence that FMS do not naturally emerge but must be intentionally taught, practiced and reinforced overtime (Logan et al. 2012) or children may decline in their FMS competency.

Although both the TGMD‐3 and MABC‐2 demonstrated internal consistency—each significantly predicting their own scores 1 year later after controlling for confounding variables (e.g., race, ethnicity, BMI, SES, childcare centre, screen time and MVPA)—there were no significant cross‐predictive relationships between the two assessments over time. That is, TGMD‐3 performance did not predict MABC‐2 scores at follow‐up, and vice versa. This lack of longitudinal association is a novel finding for the present study and suggests that these tools assess distinct constructs of FMS performance. However, future research that captures additional time points are needed to replicate these findings as measurement error, reduced power due to low study attrition and variability in developmental timing of children participating may have influenced the present study's results.

The results of the present study also highlight the importance of selecting FMS assessments based on the specific purpose and context in which they are being used. For instance, if the aim is to evaluate the qualitative aspects of movement, such as technique and coordination patterns, a process‐oriented tool like the TGMD‐3 may be most appropriate. In contrast, when the goal is to identify those at risk of developmental delay in FMS, a product‐oriented assessment like the MABC‐2 may be better suited as it can be easier to score on‐site. Although proper training is still important for administering the MABC‐2, it may be more straightforward to score in real‐time such as stopping a stopwatch or counting correct trials, compared to the more detailed nuances of evaluating performance criteria required for the TGMD‐3. However, once a delay is identified through a process‐oriented tool, administering the TGMD‐3 can offer valuable insight into how the child is performing the skill—not just whether they can complete it. This deeper understanding of movement quality can help pinpoint specific motor deficits or compensatory strategies, which can inform individualized intervention planning. Therefore, using the TGMD‐3 in conjunction with the MABC‐2 may provide a more comprehensive picture of FMS performance, especially in clinical or educational settings where both identification of delays and offering targeted support are priorities. In any setting, a balanced approach of feasibility, training, time available for assessment and purpose of assessment are all critical considerations when selecting FMS assessments.

Although this study adds to the current body of literature, it is not without limitations. The primary limitation of this study is attrition related to participant loss between Years 1 and 2. Prior to data collection in Year 2, the local area experienced excessive flooding, which led to the relocation of some families. This may have resulted in nonrandomized loss of participants, which limits the generalizability of the present study. Further, as indicated by our sensitivity analysis compared those children lost to follow‐up to those who completed follow‐up, our findings suggest that attrition was not fully random and may reflect differential loss among children with greater social disadvantage and differing health behaviour profiles. As a result, longitudinal associations may be subject to selection bias and may underestimate or overestimate true relationships if characteristics associated with follow‐up were also related to motor development outcomes. In addition, the small variance explained along with large variability seen at follow‐up indicates that other extraneous variables may explain differences in FMS performance. Future work examining access to organized activity, quality of school experiences and parent support may aid the understanding of why divergence was seen in the present study over time. Despite this, this study offers notable strengths. The racial and socioeconomic diversity of the sample adds to the heterogeneity often lacking in similar studies, enhancing the practical relevance of findings across varied populations. Additionally, the administration of both the TGMD‐3 and MABC‐2 at two time points, 1 year apart, provides an opportunity to examine the longitudinal developmental trajectories of FMS performance in the early years.

### 4.1 Conclusions

The current study found that although process‐ and product‐oriented FMS assessments show meaningful associations at ages 3–4 years, these relationships weaken substantially by age 4–5 years. This shift suggests that as children develop, the motor domains captured by each tool may begin to diverge, reflecting increasingly distinct aspects of FMS performance. Examining these patterns longitudinally during a period of rapid motor development offers important insight into how different FMS evolve in early childhood. These findings underscore the need for intentional assessment selection based on developmental stage and measurement goals, particularly in research, clinical and educational settings.

## Author Contributions

**E. Kipling Webster:** conceptualization, writing – review and editing, writing – original draft, investigation, methodology. **Katherine E. Spring:** writing – review and editing. **Justin X. Moore:** formal analysis, writing – review and editing. **Dimetrius Brandon:** writing – review and editing. **Amanda E. Staiano:** conceptualization, investigation, writing – original draft, writing – review and editing, methodology.

## Funding

This work is a pilot project of the Gulf States Health Policy Center funded by the National Institute on Minority Health and Health Disparities of the National Institutes of Health (U54 MD 008602). This work was partially supported by a NORC Center Grant # P30DK072476 entitled ‘Nutrition and Metabolic Health Through the Lifespan’ sponsored by NIDDK and by U54 GM104940 from the National Institute of General Medical Sciences of the National Institutes of Health, which funds the Louisiana Clinical and Translational Science Center. Dr. Spring is supported by the National Institute of Diabetes and Digestive and Kidney Diseases under Award No. T32DK064584. The content is solely the responsibility of the authors and does not necessarily represent the official views of the National Institutes of Health.

## Supporting information


**Table S1:** Comparison of participant characteristics between participants with (72) and without (45) follow‐up observations, within Pause & Play study.

## Data Availability

The data that support the findings of this study are available from the corresponding author upon reasonable request.
