# Supplementary material for: Diverging Paths: Longitudinal and Reciprocal Associations Between Two Fundamental Motor Skill Assessments in Preschoolers
Source: Child Care Health Dev. 2026 May 18;52:e70295. doi: 10.1111/cch.70295 (PMC13183608; doi:10.1111/cch.70295)
Supplement: Supplementary file 1 — Table S1: Comparison of participant characteristics between participants with (72) and without (45) follow‐up observations, within Pause & Play study. [file CCH-52-e70295-s001.docx]

| **Supplemental Table 1. Comparison of participant characteristics between participants with (72) and without (45) follow-up observations, within Pause & Play study.** | | | |
| --- | --- | --- | --- |
|  | **Participants with Follow-Up**  **(n = 72)** | **Participants without Follow-Up**  **(n = 45)** |  |
| **Characteristic** | **N (%) or Mean (SE^A^)** | | **p-value** |
| Mean follow-up (SE), in days | 363.3 (2.5) | - |  |
| Mean age (SE), in years | 3.25 (0.06) | 3.53 (0.08) | <0.01 |
| Gender |  |  |  |
| Female | 36 (50.0) | 26 (57.8) | 0.41 |
| Male | 36 (50.0) | 19 (42.2) |  |
| Race |  |  |  |
| Asian | 6 (8.3) | 4 (8.9) | <0.01 |
| Black | 28 (38.9) | 29 (64.4) |  |
| Other | 1 (1.4) | 3 (6.7) |  |
| White | 37 (51.4) | 9 (20.0) |  |
| Ethnicity |  |  |  |
| Hispanic or Latinx | 2 (2.8) | 2 (4.4) | 0.63 |
| Non-Hispanic/Latinx | 70 (97.2) | 43 (95.6) |  |
| Mean BMI Percentile (SE) | 57.65 (3.59) | 67.51 (3.70) | 0.07 |
| Annual Household Income |  |  |  |
| <$30,000 | 18 (25.0) | 23 (51.1) | 0.03 |
| $30,000 – 69,999 | 5 (6.9) | 4 (8.9) |  |
| $70,000 – 109,999 | 11 (15.3) | 5 (11.1) |  |
| $110,000+ | 27 (37.5) | 7 (15.6) |  |
| Not reported | 11 (15.3) | 6 (13.3) |  |
| Mean MVPA (SE), in minutes | 99.53 (4.19) | 116.51 (7.67) | 0.04 |
| Quartiles of MVPA |  |  |  |
| 1^st^ Quartile (36.50 – 81.25) | 15 (20.8) | 5 (11.1) | 0.40 |
| 2^nd^ Quartile (81.25 – 100.13) | 13 (18.1) | 9 (20.0) |  |
| 3^rd^ Quartile (100.13– 125.38) | 15 (20.8) | 6 (13.3) |  |
| 4^th^ Quartile (125.38– 226.00) | 10 (13.9) | 10 (22.2) |  |
| Missing | 19 (26.4) | 15 (33.3) |  |
| TV in bedroom |  |  |  |
| No | 43 (59.7) | 19 (42.2) | 0.07 |
| Yes | 29 (40.3) | 26 (57.8) |  |
| Mean screen time per day (SE), in minutes | 283.33 (29.7) | 415.33 (41.3) | <0.01 |
| Mean television time per day (SE), in minutes | 108.17 (8.17) | 137.33 (12.49) | 0.04 |
| Mean computer time per day (SE), in minutes | 64.62 (11.05) | 105.00 (14.12) | 0.03 |
| Mean videogames time per day (SE), in minutes | 73.45 (14.17) | 68.00 (10.27) | 0.76 |
| Mean smartphone time per day (SE), in minutes | 69.18 (9.47) | 84.32 (12.18) | 0.32 |
| Mean tablet time per day (SE), in minutes | 86.67 (10.48) | 102.16 (13.43) | 0.36 |
| ^A^ SE = Standard error | | | |
